# Assessment of the Impact of SARS-CoV-2 Infection on the Sexual Function of Women, Levels of Oxytocin and Prolactin: A Prospective Cohort Study

**DOI:** 10.3390/jcm13082230

**Published:** 2024-04-12

**Authors:** Klaudia Żak, Ernest Starek, Agnieszka Korga-Plewko, Karolina Rasoul-Pelińska, Monika Abramiuk, Mariola Michalczuk, Alicja Rajtak, Jan Kotarski, Karolina Frankowska, Liliana Bis, Marta Ostrowska-Leśko, Marcin Bobiński

**Affiliations:** 1 Department of Medical Chemistry, Medical University of Lublin, 20-059 Lublin, Poland; 2 I Chair and Department of Oncologic Gynecology and Gynecology, Medical University of Lublin, 20-081 Lublin, Poland; karolina.rasoul@gmail.com (K.R.-P.); monika.abramiuk@umlub.pl (M.A.); 61055@student.umlub.pl (A.R.); k.frankowska10@gmail.com (K.F.); marcin.bobinski@umlub.pl (M.B.); 3 Independent Medical Biology Unit, Medical University of Lublin, 20-090 Lublin, Poland; agnieszka.korga-plewko@umlub.pl (A.K.-P.); mariola.michalczuk@umlub.pl (M.M.); 4 Independent Laboratory of Minimally Invasive Gynecology and Gynecological Endocrinology, Medical University of Lublin, 20-059 Lublin, Poland; 5 Independent Laboratory of Cancer Diagnostics and Immunology, Medical University in Lublin, 20-081 Lublin, Poland; jan.kotarski@umlub.pl; 6 Chair and Department of Toxicology, Medical University of Lublin, 20-090 Lublin, Poland; marta.ostrowska-lesko@umlub.pl

**Keywords:** SARS-CoV-2, COVID-19, oxytocin, prolactin, FSFI, female sexual function

## Abstract

(1) **Background**: There is a lack of direct evidence on whether SARS-CoV-2 affects women’s sexual function through a biological-organic mechanism. Existing studies on the topic are few and have produced contradictory results. This study aims to explore the possible relationship between sex hormones and sexual function in patients who have been infected with SARS-CoV-2. Moreover, we aimed to determine whether these changes are related to the clinical course of COVID-19 and whether they are temporary or long-lasting. (2) **Methods**: A study was conducted on 104 women, including 64 women infected with COVID-19 and a control group of 40 healthy women, between January 2021 and August 2022. Blood samples were collected to measure prolactin and oxytocin levels, and a clinical assessment was performed 3 and 6 months later. Sexual function self-assessment was captured based on the FSFI scale. (3) **Results**: Our study found that patients with severe COVID-19 had better sexual satisfaction scores one month after recovery but no discernible difference after six months. High levels of serum prolactin were observed in patients with active COVID-19 but became similar to a control group after one month and remained stable over time. Higher prolactin levels were significantly associated with increased arousal and hydration. Individuals with severe COVID-19 had notably low levels of plasma oxytocin, but there was no correlation between oxytocin levels and sexual satisfaction. (4) **Conclusions**: The gynecologic symptoms, as well as disturbances in oxytocin and prolactin levels, might be observed in a short time after infection. However, SARS-CoV-2 infection has no lasting effect on sexual function, oxytocin, and prolactin levels among women.

## 1. Introduction

The coronavirus disease 2019 (COVID-19) pandemic has dramatically affected people’s lives [1,2]. Not only were their health issues and overall quality of life noticeably affected but a decline in the quality of sex life also turned out to be significant [3,4,5,6,7,8,9,10]. According to the World Health Organization, sexual health is fundamental to overall health and well-being [11]. Female sexual function is part of a woman’s self-identity and is closely related to psychological, hormonal, and physical factors [12]. The COVID-19 pandemic has affected sexual health at the individual, systemic, and societal levels [13]. Studies, including meta-analyses, have shown that during the pandemic, the general population noted a decrease in the assessment of female sexual functioning, including satisfaction, arousal, orgasm, and pain measured by Female Sexual Function Index (FSFI) [10,14,15,16,17,18,19]. Even though numerous studies analyzing the effects of the pandemic on women’s sexual function in the general population have been conducted, there is no clear evidence that the severe acute respiratory syndrome coronavirus 2 (SARS-CoV-2) infection causes sexual dysfunction through an organic mechanism in women. According to a study by Iuliano et al., women with a good sense of smell tend to have higher scores in all FSFI subscales, except for sexual desire [20]. However, researchers have agreed that some COVID-19 patients experience olfactory impairment during and after the infection. This presents an interesting hypothesis that COVID-19 patients with impaired sense of smell may suffer from negative effects on their sexual function. The short and long-term impact of COVID-19 on sexual function remains largely unexplored [21,22,23]. Moreover, the results of the studies published so far are contradictory and do not give a clear answer [22,23].

The endogenous hormones potentially having the most significant share in sexual female life are, i.a., estrogens, androgens, progesterone, prolactin, oxytocin, and glucocorticosteroids [24,25]. The levels of those hormones were also disturbed during the SARS-CoV-2 infection [26,27]. Analyzing the role of sex hormones in terms of COVID-19 infection, we have to take into account two potential general mechanisms of action: the impact of a particular hormone on the clinical course of the infection and the effect of infection on the hormonal status and indirectly on patients’ sexual function. Since 2019, multiple studies analyzing nearly every aspect of COVID-19 and its relations with multiple diseases and parameters have been published due to the efforts of a wide range of scientists and clinicians [27,28,29,30,31,32,33,34,35,36,37]. However, the available literature analysis indicates a gap in knowledge about the relationship between two hormonal factors, namely oxytocin and prolactin, this disease, and female sexual function.

Oxytocin, a hypothalamic hormone commonly referred to as the “love hormone”, plays various roles in humans, including regulating parturition and lactation, promoting closeness and intimacy, and even affecting sexual satisfaction [28,29,38,39]. Serum oxytocin positively correlates with the pleasure and intensity of orgasms of both sexes [40,41,42]. Considering the fact that oxytocin has cardiovascular protective properties and also manifests anti-inflammatory and antioxidant features, the hypothesis of the use of exogenous oxytocin in COVID-19 cases seems to be justified [28,30]. The study by Wang et al. suggests that oxytocin antagonizes the negative effects of angiotensin II, which, as we know, is a crucial enzyme in SARS-CoV-2 infection [28]. In addition, the study by Imami et al. suggests that intravenous carbetocin, an oxytocin agonist, could be used to prevent or lessen the cytokine storm associated with moderate COVID-19 cases [30]. Researchers are consistent: oxytocin may positively impact COVID-19 treatment, especially in acute or long-lasting infection [28,29,30,31,32,33,39,40,41,42]. However, to the best of our knowledge, there is no available data on the plasma levels of oxytocin in patients with acute COVID-19. This information seems crucial for further research on the potential connection between the “love hormone” and SARS-CoV-2 infection [33]. Furthermore, the correlation between oxytocin, COVID-19, and sexual function has not yet been described. Nevertheless, it remains unclear whether COVID-19 may disrupt sexual function by affecting oxytocin levels or if pre-existing imbalances in oxytocin concentration may influence infection progression and, as a consequence, worsen sexual function.

Prolactin is a peptide hormone, which, among its multiple roles, regulates sexual function and acts as a satiation hormone following sexual engagement, controlling sexual urges through feedback loops to dopaminergic neurons [34,43,44,45]. Various physiological factors may lead to a brief and minor rise in prolactin production, such as orgasm, physical activity, stress, slumber, and stimulation of the nipples [46]. There is evidence suggesting that SARS-CoV-2 infection could impact and cause prolactin levels to increase, though there remains a lack of consensus among researchers [27,34,35,36]. Prolactin has a positive impact on the immune system, promoting the release of pro-inflammatory cytokines; however, significantly elevated levels can have a negative impact by intensifying already existing inflammation [34,37]. Al-Kuraishy et al. and Sen have both proposed hypotheses suggesting that the slightly increasing prolactin concentration could potentially improve the outcomes of COVID-19 [34,37]. Therefore, it can be hypothesized that elevated prolactin levels as a result of COVID-19 disease may result in sexual function disorders.

The objective of this study was to investigate any potential correlation between sex hormone levels (prolactin, oxytocin) and sexual function self-assessment in patients who have been infected with SARS-CoV-2. The study also aimed to determine whether these changes are linked with the clinical course of COVID-19 and short and long-term variations in female genital function.

## 2. Materials and Methods

### 2.1. Study Population

The study involved 148 patients. In total, 44 patients were excluded, 42 due to non-compliance and 2 because of pregnancy during the observation period. The study analysis was conducted on a group of 104 patients. Among them, 54 patients were hospitalized due to COVID-19. A detailed study of population characteristics is found in Table 1.

Patients were recruited in the first Chair and Department of Gynecology Oncology and Gynecology in Lublin, the Chair and Department of Infectious Diseases in Lublin, and a temporary COVID-19 hospital in Lublin from January 2021 to August 2022. The study protocol was approved by the Bioethics Committee of the Medical University of Lublin (KE-0254/255/2020). 

The study group involved patients suffering from COVID-19. Inclusion criteria for the investigated group included confirmed SARS-CoV-2 infection, female gender, aged 18–50, premenopausal status, and willingness to participate with informed consent. Exclusion criteria included lack of consent, history of vascular or immune diseases, gynecological surgeries (minor interventions excluded), radio- and/or chemotherapy, and vaccination or immunomodulatory treatment within the last 30 days (excluding anti-SARS-CoV-2 vaccination, if the type and date of vaccination were known).

The control group involved patients with no positive SARS-CoV-2 test or history of symptoms of SARS-CoV-2 infection who had not suffered from SARS-CoV-2 within 6 months.

Patients were assigned for each study group based on features listed in Table 1.

### 2.2. Study Procedures

**Figure 1:**
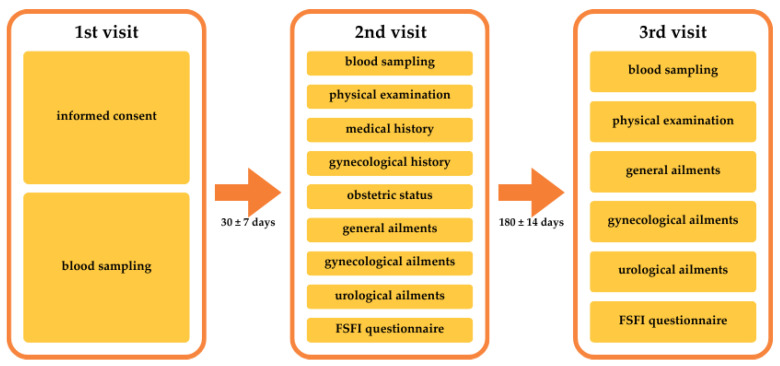
A detailed list of procedures conducted during the study.

Patients were examined at three time points: first visit, during an active infection or on the day of enrollment to the study for the control group; second visit, 30 ± 7 days after obtaining the status of convalescent or, for the control group, from the date of inclusion in the study; third visit, 180 ± 14 days after the second visit. A detailed list of procedures performed on each visit is presented in Figure 1.

### 2.3. Clinical Assessment

The patients’ medical history, including comorbidities, was collected during the second visit. Questions were asked, especially about new general symptoms that appeared after the COVID-19 disease. Special attention was paid to gynecological diseases, menstrual disorders, obstetric status, and types of delivery. Data on urological ailments, especially urinary incontinence symptoms, was also gathered. The previously collected information was confirmed during the third visit, as were the changes between the second and third visits.

### 2.4. Blood Sampling and Analysis

Blood samples were collected at every visit under standard procedures into tubes with lithium heparin and centrifuged at 3000× *g* for 10 min. Then, the plasma was separated and stored at −80 °C until further analysis. 

### 2.5. FSFI

The FSFI is a validated questionnaire used as a tool in both scientific research and clinical practice for evaluating sexual function in women [48]. It consists of 19 questions that cover a range of aspects related to sexual function, including sexual desire, arousal, lubrication, orgasm, and sexual satisfaction. The questionnaires were scored according to the standard system described by Meston et al. [49]. Scores are tallied, with a range of 1.2 to 36.0 and an optimal cutoff value of 26.0, indicating better sexual functioning [49,50]. 

### 2.6. Oxytocin and Prolactin Assessment

According to the manufacturer’s instructions, plasma oxytocin levels were determined using the RayBio Oxytocin Enzyme Immunoassay (EIA) Kit (RayBiotech Life, Peachtree Corners, GA, USA). Briefly, the samples and standards were spiked with a biotinylated oxytocin peptide and then put on the plate. Endogenous oxytocin competed for binding to the anti-oxytocin antibody with the biotinylated oxytocin peptide. Then, biotinylated oxytocin interacted with horseradish peroxidase (HRP)-streptavidin, catalyzing a color reaction. The intensity of the colorimetric signal was proportional to the amount of biotinylated oxytocin peptide and inversely proportional to the amount of endogenous oxytocin in the standard or samples.

Due to the test’s commercial prevalence, plasma prolactin level determination was performed by an external, certified laboratory using the chemiluminescence immunoassay (CLIA) method (CL-2000i, Mindray Medical, Warsaw, Poland). The analysis did not include three patients on ethinylestradiol, which impacts plasma prolactin levels.

### 2.7. Statistical Analysis

Statistical analysis was performed using Statistica 13 software (StatSoft, Krakow, Poland). The normality of the variable distribution was verified using the Shapiro–Wilk test. The concentrations of prolactin and oxytocin were measured three times in patients’ serum at different time points and analyzed using one-way analysis of variance for repeated measurements and Tukey’s honest significant difference test. Data were presented as mean ± standard error (SE).

The number of patients with sexual dysfunction in each study group was compared using the Chi-square or Fisher’s exact test. The Spearman rank correlation coefficient was used to evaluate the relationship between FSFI questionnaire answers and hormone concentrations.

## 3. Results

### 3.1. Clinical Assessment

Our analysis showed that following recovery from COVID-19, patients continued to report a range of general disorders for up to 30 days, with statistically significant numbers experiencing chronic coughs, dyspnoea, tachycardia, weakness, and issues with smell, taste, concentration, and memory loss (*p* ≤ 0.05). A total of 52.0% of patients with severe COVID-19 and 45.0% with mild or moderate illness reported these symptoms. Even after 180 days, these general disorders remained significant (*p* ≤ 0.05), with 29.0% of severely ill patients and 12.0% of those with mild or moderate illness reporting such symptoms.

**Figure 2:**
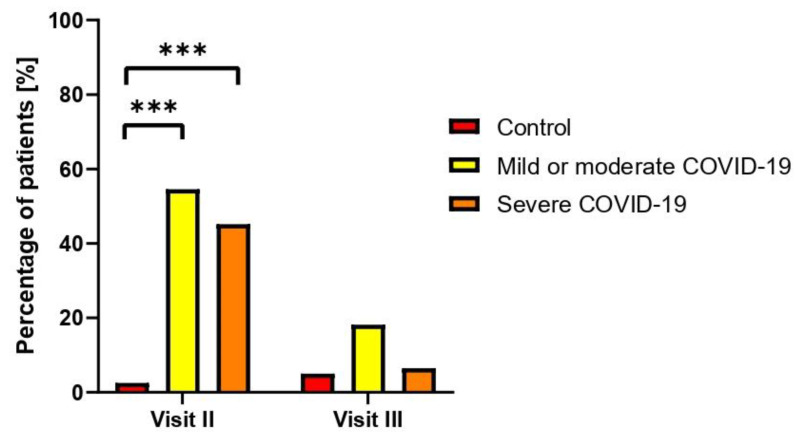
Reported gynecological disorders among control, mild or moderate COVID-19, and severe COVID-19 groups during second and third visits. *** *p* ≤ 0.001 vs. control group.

During the second visit, the study revealed a statistical significance (*p* ≤ 0.001) in gynecological issues, such as menstrual disorders, including metrorrhagia, dysmenorrhea, hypermenorrhoea, or menometrorrhagia, among patients with severe COVID-19 and those with mild to moderate illness compared to a control group of women (Figure 2). However, by the third visit, there was no significant difference between the groups.

During the second visit, newly reported urological disorders, such as pollakiuria and stress urinary incontinence, were observed, with no significant difference between the control group (0.0%) and the groups of severely ill (10.0%) and mild to moderately ill (6.0%) women. By the third visit, there was still no significant difference between the groups, with 6.0% of severely ill women, 0.0% of those with mild or moderate illness, and 2.5% of patients without a history of COVID-19 reporting urological disorders.

### 3.2. FSFI Evaluation

As noted above, FSFI was filled 30 days ± 7 after entering the study (second visit). The study showed that 30 days after entering the study (second visit), patients with severe COVID-19 had the highest level of FSFI total score among all groups (mean 26.46 ± 4.81) compared to mild or moderate COVID-19 groups (mean 25.91 ± 7.79) and the control group (mean 24.71 ± 9.01). At 180 days ± 14 after the inclusion into the study (third visit), the highest FSFI total score was observed in patients from the control group (mean 27.22 ± 7.73), compared to the group of mild or moderate COVID-19 (25.63 ± 7.35) and the group of severe COVID-19 (26.98 ± 4.27) (Table 2). The most significant change in the FSFI total score between two visits was observed in the control group. In this group, the FSFI total score increased by 2.51 from 24.71 (second visit) to 27.22 (third visit). In the groups where patients suffered from COVID-19, the change was lower. At second visit, 36.36% (4/7) of patients with severe COVID-19, 50.0% (6/12) of patients with mild or moderate COVID-19, and 33.33% (8/24) of the control group had means of FSFI score of 26 or less, indicating the presence of significant clinical sexual dysfunction. There was no between-groups statistical significance noted (*p* = 0.62). At the third visit, 16.67% of patients with severe COVID-19, 50.0% of patients with mild or moderate COVID-19, and 35.71% of the control group had mean FSFI scores of 26 or less, and there was no statistical between-groups significance noted (*p* = 0.44).

### 3.3. Oxytocin

**Figure 3:**
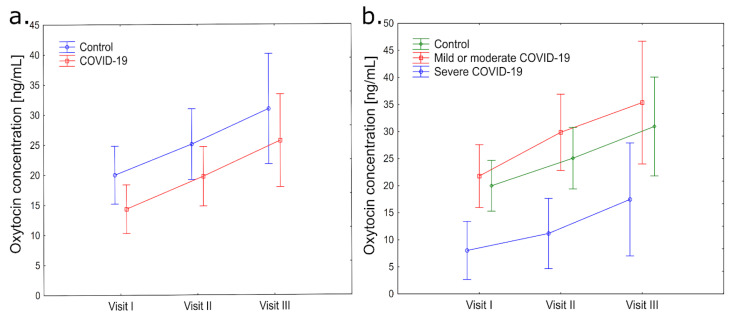
(**a**) Changes in oxytocin concentration levels in the blood plasma of healthy patients and patients after COVID-19 infection during three subsequent visits (first visit, second visit, and third visit); (**b**) changes in oxytocin concentration levels in control, mild or moderate COVID-19, and severe COVID-19 groups were measured at three-time points (first visit, second visit, and third visit).

The concentration of oxytocin was lower in the COVID-19 groups at all time points (Figure 3a) compared to the control group. During the first measurement of oxytocin, it was observed that the COVID-19 group had statistically significantly (*p* = 0.03) lower levels of oxytocin concentrations than the non-COVID-19 group (first visit: 10.28 ± 2.88 ng/mL vs. 22.76 ± 5.09 ng/mL). At the second and third time points, levels of oxytocin were also lower but without statistical significance (*p* = 0.08) (second visit: 14.98 ± 4.41 ng/mL vs. 28.60 ± 5.75 ng/mL; third visit: 19.19 ± 4.28 ng/mL vs. 36.10 ± 10.48 ng/mL) (Figure 3a).

Patients in the mild or moderate COVID-19 group had the highest levels of oxytocin concentration during each visit (first visit: 21.78 ± 8.28 ng/mL; second visit: 29.87 ± 10.32 ng/mL; third visit: 35.36 ± 18.14 ng/mL), with the lowest levels observed in the severe COVID-19 group (first visit: 8.02 ± 1.96 ng/mL; second visit: 11.16 ± 2.98 ng/mL; third visit: 17.45 ± 5.40 ng/mL) compared to the control group (first visit: 19.98 ± 4.70 ng/mL; second visit: 25.09 ± 5.33 ng/mL; third visit: 30.94 ± 6.93 ng/mL) (Figure 3b). According to the research findings, there was no significant difference in the distribution of oxytocin concentrations among the study groups. However, all groups noticed a noticeable increase during subsequent time points.

No statistically significant variations were found in the overall FSFI total score and oxytocin outcomes among the study groups during subsequent visits. However, it was discovered that the level of oxytocin during second visit correlated with FSFI questionnaire answers: satisfaction in the control group (*p* = 0.016). During third visit, an inverse relationship between oxytocin and excitement was observed in patients in the mild or moderate COVID-19 group (*p* = 0.018).

### 3.4. Prolactin

**Figure 4:**
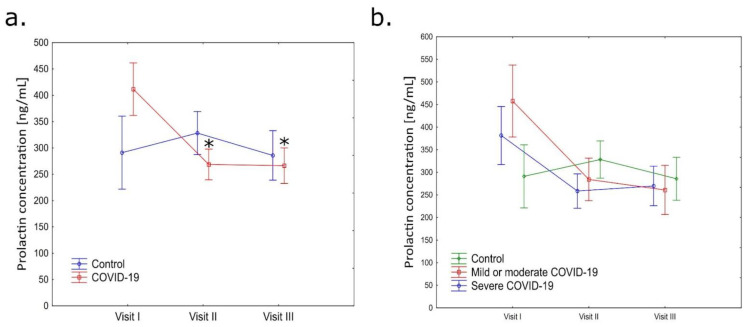
(**a**) Changes in prolactin concentration level in the blood plasma of healthy patients and patients after COVID-19 infection during three subsequent visits (first visit, second visit, and third visit); (**b**) changes in prolactin concentration levels in control, mild or moderate COVID-19, and severe COVID-19 groups were measured at three time points (first visit, second visit, and third visit). * *p* ≤ 0.05 vs. first visit in COVID-19 group.

During the first visit, patients in the active phase of COVID-19 had noticeably elevated levels of prolactin in comparison to healthy patients (first visit: 422.60 ± 53.18 ng/mL vs. 283.48 ± 32.75 ng/mL) (Figure 4a). In subsequent visits, these levels decreased to levels comparable to the control group (second visit: 244.87 ± 23.17 ng/mL vs. 333.85 ± 33.70 ng/mL; third visit: 278.97 ± 37.44 ng/mL vs. 264.47 ± 39.84 ng/mL). However, there were no statistically significant differences in prolactin concentrations between the study groups at specific time points. A significant difference was observed inside the group between prolactin concentration levels during the second and third visits compared to the first in the COVID-19 group (*p* ≤ 0.05) (Figure 4a).

The mild or moderate COVID-19 group had the highest level of prolactin concentration among all groups during the first visit (466.82 ± 101.79 ng/mL), while the severely ill patients had a mean prolactin concentration of 335.24 ± 43.48 ng/mL (Figure 4b). The lowest prolactin concentration was in the control group, with a mean of 298.60 ± 39.97 ng/mL. At second visit, the highest prolactin level was observed in the control group, with a mean of 324.30 ± 35.40 ng/mL. The mild or moderate ill patients group had a mean of 266.24 ± 38.49 ng/mL. The lowest prolactin concentration at this visit was in a group of severe COVID-19 patients (237.40 ± 28.06 ng/mL). At third visit, which was 180 days after entering the study, the levels of prolactin were comparable in all groups (severe COVID-19: 275.74 ± 36.33; mild or moderate COVID-19: 260.85 ± 68.30; control: 275.0 ± 50.29 ng/mL) (Figure 4b).

A significant correlation between FSFI answers and prolactin level concentrations has been found. In the mild or moderate group, there was a significant correlation between hydration and prolactin concentration during second visit (*p* = 0.040). In contrast, in patients with severe COVID-19, prolactin levels during third visit showed an inverse correlation (*p* = 0.022) with excitement, hydration (*p* = 0.004), and FSFI total score (*p* = 0.030). Additionally, in the control group, prolactin levels during the same time point showed an inverse correlation (*p* = 0.037) with satisfaction and pain (*p* = 0.006).

## 4. Discussion

Nowadays, it is difficult to recall the scale of how COVID-19 changed our lives roughly three years ago. In the beginning, we were worried about our physical health, but with time, we started to think also about our mental health. We, as physicians, asked ourselves if and how SARS-CoV-2 may also affect sexual functions. The results in this aspect are not consistent. To begin with, we should not forget that female sexual dysfunction was already a prevalent issue in 30–50% of women before the pandemic, although it may not have been widely recognized, which is crucial when considering the impact of the pandemic on women’s sexual function [51]. Two of the biggest meta-analyses, performed by Hessami et al. and Qaderi et al., showed that sexual function assessments during the COVID-19 pandemic have significantly decreased compared to pre-pandemic times [17,18] Only three studies considered the impact of SARS-CoV infection on this crucial aspect of human life. According to Gencer et al., women who were treated at home for COVID-19 had a higher risk of developing female sexual dysfunction (FSD) compared to healthy women [21]. There were only two studies that analyzed the sexual functions of women who had been infected with SARS-CoV-2 over time. One of these studies, conducted by Nawaz et al., showed a significant decrease in the FSFI score during the 60-day follow-up period after infection compared to pre-infection [22]. On the contrary, the second study found no impact of the disease on the overall FSFI score [23]. However, compared to our study, none of the above-mentioned studies assessed long-term effects on female sexual functions.

The relationship between age, clinical characteristics, and the severity of COVID-19 has been extensively studied, and our research confirms these findings [52,53,54,55,56]. Numerous studies have shown that patients with COVID-19 may experience reduced libido and gynecological and urological complaints [57,58,59]. According to researchers, almost half of the patients reported changes in their menstrual cycle, including worsened symptoms, changes in menstrual volume, and longer cycles [57,60]. Our study confirms these findings. However, we have also observed that these symptoms tend to disappear over time.

Based on our long-term observation, we have found that any decline in sexual function among women is only temporary and does not have a lasting impact. To the best of the authors’ knowledge, before conducting present study, no studies were found that differentiate the impact of COVID-19 severity on women’s sexual function. Our study indicates that the severity of the course of COVID-19 does not affect the assessment of sexual function in long-term observation. The patients with severe illness recorded the highest FSFI total score 30 days after being included in the study. This could be attributed to the stress caused by the illness and extended hospital stay, which resulted in prolonged separation from their partners. Women’s sexual needs are greatly influenced by their emotional requirements [61]. After 180 days of participation in the study, the results of FSFI were comparable across all groups. While SARS-CoV-2 infection may have an impact on women’s sexual function in the short term, we have not observed any persistent disorders in the long term.

Moreover, we tried to measure more objective indicators of sexual functions; therefore, we chose two hormones, prolactin and oxytocin, and checked their concentrations at three-time points. Our study showed that patients actively infected by SARS-CoV-2 have significantly higher serum prolactin levels. The outcome of our research is consistent with existing studies, though it is worth noting that there are divergent findings in this field [27,35,36]. However, after a month, these levels return to normal in recovering patients and remain stable during follow-up, regardless of the severity of their COVID-19 course. While there was no initial correlation found between prolactin levels and sexual function, a long-term follow-up of COVID-19 patients indicated a significant relationship between higher prolactin levels and increased arousal and hydration, as measured by the FSFI.

Studies have shown that prolactin can boost the immune system by increasing the release of pro-inflammatory cytokines, potentially causing immunoinflammatory disorders [34,37]. Researchers have hypothesized that raising prolactin levels, a hormone involved in the immune system, may improve COVID-19 outcomes [34,37]. On the other hand, some studies have shown contradictory results that high serum prolactin may worsen disease severity depending on the phase of SARS-CoV-2 infection [34,62]. The relationship between prolactin and COVID-19 severity may also be affected by other factors such as age and patients’ underlying health conditions. Based on the findings, hyperprolactinemia in individuals with COVID-19 may be a result of stress since there was no correlation between serum prolactin levels and the severity of COVID-19 when other endocrinopathies were not present [34].

Recent studies have suggested that oxytocin might have several potential mechanisms that could assist in the effective and affordable treatment and prevention of COVID-19 [28,29,30,31,32,33]. Despite many potential mechanisms by which oxytocin could be used in the fight against the SARS-CoV-2 virus, studies beyond animal models have not yet been conducted. There is also currently no data on plasma oxytocin concentrations in patients during the acute phase of COVID-19. Also, the potential correlation between fluctuations in plasma oxytocin levels and the severity of COVID-19, which could suggest other disease states caused by lowering its concentration, has not been determined [33].

Our research has revealed for the first time that patients who suffered from a severe case of COVID-19 had significantly reduced levels of plasma oxytocin throughout their illness. Furthermore, the long-term monitoring of oxytocin levels has indicated a persistent decline in patients who have recovered from COVID-19 in comparison to those who have not. This underscores the importance of investigating long-term COVID mechanisms and the potential use of oxytocin as a preventative or treatment measure. 

The relationship between oxytocin and COVID-19 severity is complex and may involve various factors, including individual differences in oxytocin levels, the stage of the disease, and other underlying health conditions. Even though the group of patients with severe COVID-19 had lower levels of oxytocin, there was no connection found with sexual satisfaction after conducting statistical analysis, irrespective of the measurement time and the severity of the disease. This implies that the mechanisms responsible for decreasing serum oxytocin levels in patients do not impact their sexual function. 

However, the influence of oxytocin on women’s sexual lives seems to warrant further investigation, given that certain challenges were encountered while analyzing the results of the oxytocin study. 

The analysis of the oxytocin study results faced particular challenges. Due to freezing samples and analyzing all oxytocin tubes together after the study ended, higher oxytocin concentrations during subsequent visits in all study groups may have resulted from degradation over time, lowering concentrations in earlier tubes. This outcome is evident in all groups. Moreover, the lack of cut-off points for oxytocin concentrations hindered the analysis of the determinations.

It is important to underline that our study had some limitations. Firstly, patients were recruited during a challenging and uncertain period of the pandemic, and the number of cases in the population varied throughout the study due to the nature of COVID-19, which occurs in waves. Well-established risk factors, such as age and parity, impacted the outcome. Recruiting and retaining study participants was affected by the COVID-19 pandemic, as the fluctuating health, social, and economic conditions made it challenging. One of the study’s main limitations was the relatively small group of patients. The challenging pandemic period we all experienced contributed to this limitation, as it posed numerous obstacles at every study stage. The fluctuating wave-like nature of the COVID-19 pandemic also resulted in instability. It ultimately affected our research results, including the evaluation of sexual satisfaction and prolactin and oxytocin levels due to the inability to test hormone levels in a specific phase of the cycle. Many patients also presented with cycle disorders, which further complicated the timing of hormonal sampling. Moreover, another limitation of our study is the lack of complete hormonal panel measurements, meaning the concentrations of estrogens, androgens, progesterone, and glucocorticosteroids [24,25]. Our selection of oxytocine and prolaction was primarily based on the potential connection with SARS-CoV-2 infection and, secondly, their role in female sexual functions. However, the full hormonal panel would provide more insight into COVID’s impact on female sexual functions and a better understanding of the issue. Additionally, it should be noted that the concentration of endogenous oxytocin and prolactin may be affected by various physiological factors, including gender, age, time of day, sleep, and medications [63].

It is crucial to note that our study excluded patients with sexual dysfunctions, lack of sexual activity, use of libido-reducing drugs in the last three months, personality disorders, or other mental illnesses (such as depression) from the FSFI questionnaire assessment. This factor may have affected the study’s results compared to studies that rely solely on the collected FSFI questionnaire without a thorough interview and physical examination. It should be noted that the FSFI assessment has limitations as it does not consider all aspects of sexual function, including emotional, relational, or social factors, that may impact a woman’s sexual quality of life. Furthermore, the FSFI primarily focuses on the physiological elements of sexual function. As a self-assessment, the FSFI scale’s answers are subjective and may be influenced by individual interpretations, leading to potential bias in the results. Additionally, women may answer the FSFI questions in a way that aligns with societal or research expectations, further skewing the results. The scale also evaluates sexual function in isolation without considering the broader context of a woman’s sexual life, such as emotional connections or psychological concerns that may affect sexual well-being.

## 5. Conclusions

Based on our longitudinal observation, we have found that SARS-CoV-2 infection can lead to gynecological problems that can be resolved over time. However, it does not cause any long-lasting damage to the sexual function of women. Additionally, it was observed that those who had a severe manifestation of COVID-19 exhibited significantly reduced concentrations of plasma oxytocin throughout the course of their disease. Moreover, long-term observations of oxytocin levels have indicated a persistent decrease in patients who have recovered from COVID-19 compared to those who have not. Those changes in the oxytocin levels highlight the need to investigate the mechanisms involved in long-term COVID-19 and the potential role of oxytocin as a preventive and treatment measure. The least explored is the connection of prolactin with COVID-19 disease. Elevated levels of blood prolactin were observed in patients diagnosed with active COVID-19. However, after one month, when patients became convalescent, their prolactin levels were comparable to the control group and remained stable during follow-up, regardless of the severity of their COVID-19 course. This finding provides evidence of a significant association between prolactin and the systemic inflammatory response induced by SARS-CoV-2. However, it is noteworthy that the prolactin levels eventually revert back to their initial baseline.

## Figures and Tables

**Table 1:** A detailed description of the division and inclusion criteria for the groups used in the study and their characteristics.

Study Group	Control	Mild or Moderate COVID-19		Severe COVID-19
COVID-19 status	No history of COVID-19 patients	COVID-19 patients		
NIH group [47]	n/a	1. mild illness	2. moderate illness	3. severe illness
Criteria for assignment	No positive SARS-CoV-2 test or history of symptoms of SARS-CoV-2 infection, and no SARS-CoV-2 within 6 months	Any symptom of COVID-19 *, or abnormal chest imaging	Evidence of lower respiratory disease during clinical assessment or imaging, and SpO_2_ ≥ 94%	SpO_2_ < 94%; PaO_2_/FiO_2_ < 300 mm Hg; RR > 30 breaths/min, or LI > 50%
*n*	40	33		31
Age (years)	30 ± 8	34 ± 8		43 ± 7
At least one childbirth in anamnesis	45.0%	64.52%		80.65%
Average number of pregnancies	0.66	1.39		1.97

*, e.g., fever, cough, sore throat, malaise, headache, muscle pain, nausea, vomiting, diarrhea, loss of taste and smell) but not shortness of breath, dyspnea, or positive SARS-CoV-2 test. LI, lung infiltrates; n/a, not applicable; PaO_2_/FiO_2_, a ratio of arterial partial pressure of oxygen to a fraction of inspired oxygen; RR, respiratory rate; SpO_2_, pulse oximetry.

**Table 2:** Changes in FSFI scores of healthy patients and patients with mild or moderate and severe COVID-19 infection during three subsequent visits.

FSFI	Second Visit			Third Visit		
	Control (*n* = 40)	Mild or Moderate COVID-19 (*n* = 31)	Severe COVID-19 (*n* = 33)	Control (*n* = 40)	Mild or Moderate COVID-19 (*n* = 31)	Severe COVID-19 (*n* = 33)
Total Sexual Function Score	24.71 ± 9.01	25.91 ± 7.79	26.46 ± 4.81	27.23 ± 7.73	25.63 ± 7.35	26.98 ± 4.27
Desire	3.73 ± 1.45	3.82 ± 1.39	3.36 ± 0.99	4.07 ± 1.38	3.60 ± 1.31	3.44 ± 0.94
Arousal	4.03 ± 1.70	4.18 ± 1.46	4.26 ± 0.87	4.69 ± 1.35	4.08 ± 1.38	4.56 ± 1.19
Lubrication	4.40 ± 1.87	5.01 ± 1.54	5.12 ± 1.03	4.67 ± 1.87	4.88 ± 1.41	5.34 ± 0.84
Orgasm	4.42 ± 1.83	4.39 ± 1.59	4.56 ± 1.08	4.57 ± 1.45	4.18 ± 1.54	4.59 ± 0.96
Satisfaction	4.67 ± 1.77	4.77 ± 1.22	4.77 ± 0.71	5.14 ± 1.38	4.50 ± 1.34	4.88 ± 0.58
Pain	3.46 ± 2.20	4.10 ± 1.67	4.39 ± 1.57	4.08 ± 1.40	4.40 ± 1.49	4.17 ± 1.47
Female Sexual dysfunction	33.33%	50.0%	36.36%	35.71%	50.0%	16.67%

## Data Availability

The data presented in this study are available on request from the corresponding author.
